# Optimization of Radiation Protection in Pediatric Interventional Radiology in Latin America and the Caribbean: Development, Advancements, Challenges and Achievements of the OPRIPALC Program

**DOI:** 10.3390/children10121858

**Published:** 2023-11-27

**Authors:** Carlos Ubeda, Elise Vano, María Perez, Pablo Jimenez, Emilie van Deventer, Raúl Ramirez, Alejandro Nader, Patricia Miranda

**Affiliations:** 1 Departamento de Tecnología Médica, Facultad de Ciencias de la Salud, Universidad de Tarapacá, Arica 1000000, Chile; 2 Radiology Department, Faculty of Medicine, Complutense University, IdIS, San Carlos Hospital, 28040 Madrid, Spain; eliseov@med.ucm.es; 3 World Health Organization (WHO), 1202 Geneva, Switzerlandvandeventere@who.int (E.v.D.); 4 Pan American Health Organization (PAHO), Washington, DC 20037, USA; jimenezp@paho.org; 5 International Atomic Energy Agency (IAEA), 1220 Vienna, Austria; r.ramirez@iaea.org (R.R.); alejandro.nader@miem.gub.uy (A.N.); 6 Luis Calvo Mackenna’s Hospital, AntonioVaras 360, Santiago 7500000, Chile; jmiranda@calvomackenna.cl

**Keywords:** radiological protection, optimization, interventional procedures, pediatrics, diagnostic reference levels

## Abstract

This article presents the development, advancements, challenges and achievements of the “Optimization of Protection in Pediatric Interventional Radiology in Latin America and the Caribbean” (OPRIPALC) program. This international initiative is led by the World Health Organization, the Pan American Health Organization and the International Atomic Energy Agency. The main objectives of OPRIPALC are to foster a culture of radiological protection in pediatric interventions, enhance these procedures’ quality, and define optimization strategies such as the use of diagnostic reference levels (DRLs). Currently, 33 centers from 12 countries participate actively in the program. Significant progress has been made towards the proposed objectives, overcoming the challenges posed by the COVID-19 pandemic. Through many virtual meetings for coordination, planning, training and follow-up, a comprehensive set of DRLs for both diagnostic and therapeutic procedures, categorized by weight and age, have been established and are in use. A consensus document on good practices is in the final stage of development. The program’s continuation into at least a second phase is essential to address pending issues, including the integration of automatic dose management systems, the levels of occupational radiation doses, their correlation with pediatric patient doses, and strategies to reduce them.

## 1. Introduction

Fluoroscopy-guided interventional procedures, and particularly cardiac catheterizations, have been increasingly used for diagnostic and therapeutic interventions, thereby reducing the use of invasive surgery. However, radiation exposure remains a major concern, especially in pediatric patients who may undergo one or several fluoroscopy-guided cardiac catheterizations during childhood, as the risk of cancer is known to be higher than in adults [1,2,3,4,5,6,7,8].

To minimize the risk of inducing cancer in children, it is essential to keep the radiation doses of pediatric X-ray procedures as low as possible while still preserving appropriate diagnostic image quality [9]. To that end, it is necessary to implement a comprehensive quality assurance program that includes, among other aspects, quality control tests of X-ray equipment, continued training of personnel in radiological protection and periodic audits of radiation doses and of the quality of patient images [10,11,12].

The International Basic Safety Standards for Protection against Ionizing Radiation and Safety of Sources (BSS) [11], which were endorsed by the Pan American Health Organization (PAHO), include specific requirements for medical exposures of patients and pay special attention to care for pediatric patients [13]. The “Bonn Call to Action” jointly developed by the International Atomic Energy Agency (IAEA) and the World Health Organization (WHO) also included recommendations aligned with these requirements [14], and the International Commission on Radiological Protection (ICRP) indicates that measures must be adopted with the aim of preventing, as far as possible, unnecessarily high doses during medical exposures to patients [1].

As part of the radiation protection system, the ICRP no dose limits or dose restrictions are recommended for individual patients because they may reduce diagnostic efficiency, causing more harm than benefit [1]. Therefore, emphasis should be placed on the justification of radiological examinations and on the optimization of radiation protection, including the use of diagnostic reference levels (DRLs) [12]. Diagnostic reference levels (DRLs) have proven to be an effective tool that helps optimize radiological protection in medical exposure of patients for diagnostic and interventional procedures [15,16,17,18,19,20,21]. However, the use of DRLs in fluoroscopy-guided cardiac interventional procedures in pediatric patients has been implemented in only a few countries [9,22,23,24,25,26,27] and not yet at a regional level.

To address this need, the WHO and the PAHO in cooperation with the IAEA implemented a program called “Optimization of Protection in Pediatric Interventional Radiology in Latin America and the Caribbean” (OPRIPALC; acronym in Spanish language) to support countries of Latin America and the Caribbean towards the achievement of those standards of safety and quality. In addition to establishing regional DRLs, OPRIPALC has the following objectives: (1) promote the culture of radiological protection in pediatric interventions, (2) improve the quality of these procedures in the participating centers, (3) define optimization strategies based on the determination and use of DRLs in a sample of hospitals representative of various countries in Latin America and the Caribbean and (4) produce a consensus document for the region that offers guidance to improve the optimization of radiation protection on fluoroscopy-guided interventions in pediatrics. The purpose of this article is to describe the development, progress, challenges and achievements of the OPRIPALC program.

## 2. Development and Participants of OPRIPALC

During 2018, countries in the Latin America and the Caribbean region (Spanish-, Portuguese-speaking), were officially invited through the PAHO country offices to participate in this international initiative.

Subsequently, a set of representative hospitals from Latin America and the Caribbean were selected, as shown in Table 1, and a series of technical coordination meetings were held to agree on a methodology and a work plan, as well as to follow-up of the committed activities. At least a dozen training activities in radiological protection and quality assurance for professionals have also been carried out. Throughout these years of work, communication has been predominantly virtual, both synchronously and asynchronously. Examples of the latter are systematic communications via email, virtual meetings or a WhatsApp group. A web page has been created and continuously updated, which has basic information about the program, the list of participating centers, the number of procedures collected, a section with relevant bibliography and supporting resources [28,29,30].

To gather technical and scientific information from each center, five surveys have been performed so far. The first survey requested information about the characteristics of each center in terms of its X-ray equipment, personnel, number of procedures performed and impact of the COVID-19 pandemic. The second survey aimed to start collecting patient dose data. The objective of the third survey was to determine the state of radiological protection of each center and obtain information to identify needs of training. The fourth survey aimed to know the quality control tests carried out in each center. The last survey aimed to gather what optimization strategies were applied in each center (all surveys can be downloaded from Annex 1, https://opripalc.org/documentos/ (accessed on 1 September 2023).

Working groups were also formed among the professionals participating from the centers with the objectives of the following: (1) developing a list of basic quality control tests; (2) harmonizing a common nomenclature to classify pediatric cardiology interventional procedures; (3) proposing levels of complexity of pediatric cardiology interventional procedures; (4) developing a standard research protocol to be distributed among the centers that required authorization from their corresponding ethics committees; (5) preparing a consensus document containing practical guidance for optimizing radiological protection and safety in pediatric interventional procedures.

Initially, the collection of patient dose values was carried out manually by each participating center and collected by the University of Tarapaca (Chile) for safekeeping and analysis. As of 2021, an automatic dose management system was used thanks to the support of the Medical Physics service of the San Carlos hospital in Madrid [29,30].

A series of scientific and dissemination papers have been prepared to be presented in scientific journals, seminars and congresses.

## 3. Main Results of OPRIPALC

### 3.1. Participation of Centers and Countries

**Figure 1:**
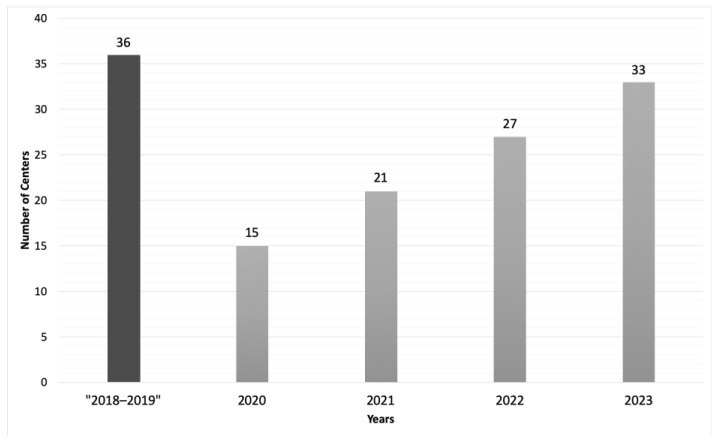
Evolution of the number of centers per year.

At the beginning of the program, in the years 2018–2019, a total of 36 centers belonging to 10 countries expressed a positive response to the invitations. Of these, 18 centers from 9 countries completed the application phase of the first two surveys in 2020. Thus, after a review process of the received data, the participation was reduced to 15 centers from the following 9 countries: Argentina, Brazil, Chile, Colombia, Costa Rica, Ecuador, Mexico, Peru and Uruguay. In 2021, 6 additional centers were confirmed, making a total of 21 hospitals from the countries mentioned above and Cuba. In the year 2022, participation increased to 27 hospitals from 11 countries (with the addition of Panama). As of 2023, 33 centers from 13 countries (including Guatemala and Venezuela) are participating. Figure 1 shows the evolution of the number of centers per year.

### 3.2. Impact of the COVID-19 Pandemic in the Centers OPRIPALC

Table 2 summarizes the most frequent responses sent by the centers as part of the first survey applied on the impact of the COVID-19 pandemic.

### 3.3. Collection of Patient Dose Data and DRLs

**Figure 2:**
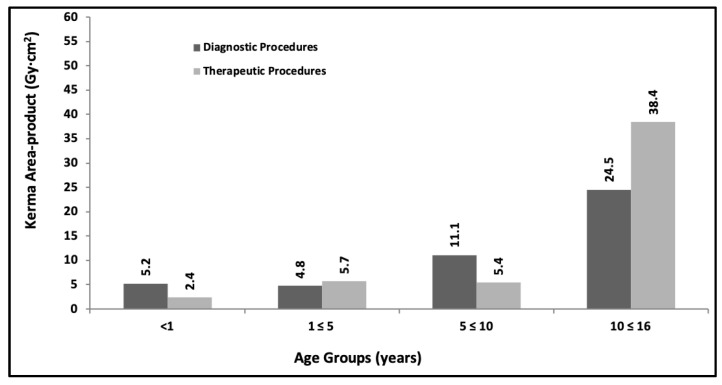
Third quartile values for kerma area-product for diagnostic and therapeutic procedures. The data are for the <1 yr, 1 to ≤5 yr, 5 to ≤10 yr and 10 to ≤16 yr age groups. Year 2020.

Regarding the collection of patient dose data and the proposal of initial DRLs, Figure 2 shows a summary of third quartile values for kerma area-product for 128 procedures collected during 2020, of which 63 were diagnostic and 65 were therapeutic, grouped by age ranges (<1 year; 1 ≤ 5 years; 5 ≤ 10 years and 10 ≤ 15 years).

**Figure 3:**
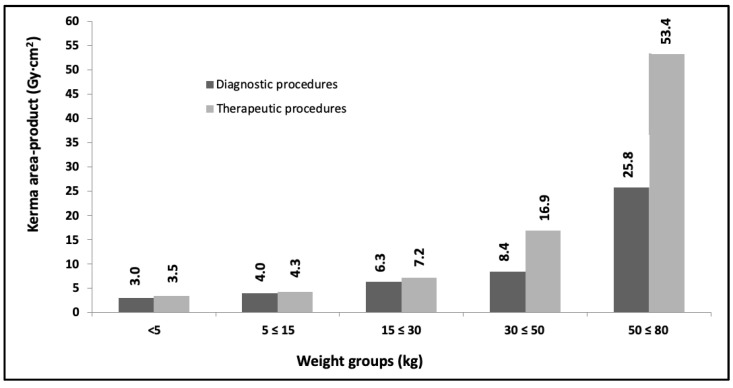
Third quartile values for kerma area-product for diagnostic and therapeutic procedures. The data are for the <5 kg, 5 to ≤15 kg, 15 to ≤30 kg, 30 to ≤50 kg and 50 to ≤80 kg weight groups. Year 2021.

**Figure 4:**
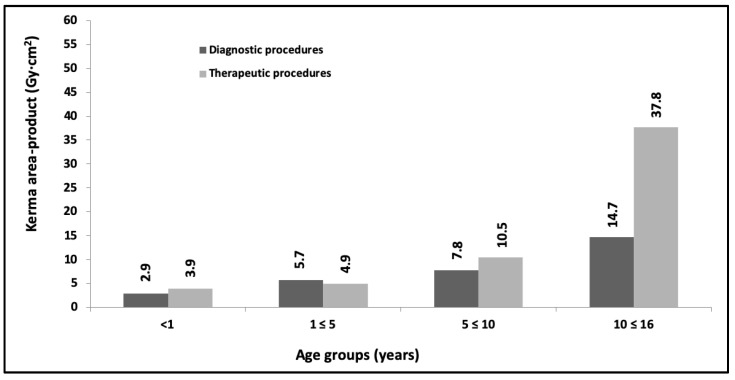
Third quartile values for kerma area-product for diagnostic and therapeutic procedures. The data are for the <1 yr, 1 to ≤5 yr, 5 to ≤10 yr and 10 to ≤16 yr age groups. Year 2021.

Figure 3 and Figure 4 show the DRLs grouped (third quartile values for kerma area-product) for the same age ranges as the previous year and weight ranges (<5 kg; 5 ≤ 15 kg; 15 ≤ 30 kg; 30 ≤ 50 kg and 50 ≤ 80 kg) for the year 2021, which corresponded to 968 procedures (268 diagnostic and 427 therapeutic).

**Figure 5:**
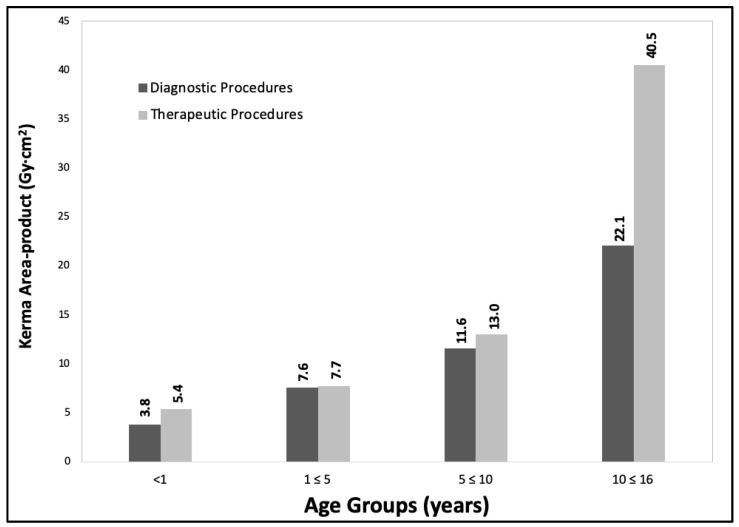
Third quartile values for kerma area-product for diagnostic and therapeutic procedures. The data are for the <1 yr, 1 to ≤5 yr, 5 to ≤10 yr and 10 to ≤16 yr age groups. Year 2022.

**Figure 6:**
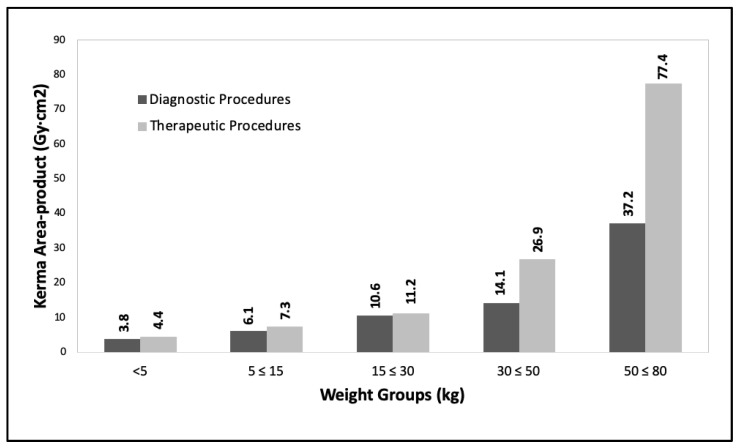
Third quartile values for kerma area-product for diagnostic and therapeutic procedures. The data are for the <5 kg, 5 to ≤15 kg, 15 to ≤30 kg, 30 to ≤50 kg and 50 to ≤80 kg weight groups. Year 2022.

During the year 2022, data from 1945 procedures were collected. Not all the requested variables were informed by the centers and, therefore, the final number of validated samples was 1587 (for the procedures grouped between diagnostic and therapeutic). Figure 5 and Figure 6 show as DRLs the third quartile values for kerma area-product obtained during this year for the same previous age and weight ranges (only results from validated and analyzed procedures are shown).

### 3.4. Dissemination of Scientific Results

As part of the dissemination activities, presentations were made at relevant scientific events (European Congress of Radiology, Vienna, Austria 2020 and 2021; XXV Brazilian Congress of Medical Physics, Sao Paulo, Brazil. 2020; Latin-American Society of Interventional Cardiology (SOLACI) Buenos Aires, Argentina, 2021; Argentine Congress of Diagnostic Imaging (CADI), Buenos Aires, Argentina, 2021; Research Seminar University of La Frontera, Temuco, Chile (2021); Third Latin American Week of Radiology, Bogota, Colombia (2021); 15th International IRPA Congress (IRPA15, Seoul, Republic of Korea, 2021; International Conference on Occupational Radiation Protection: Strengthening Radiation Protection of Workers Twenty Years of Progress and the Way Forward, Vienna, Austria, 2022; IAEA Technical Meeting on Radiation Protection in Fluoroscopically Guided Interventional Procedures, Vienna, Austria, 2022) [31,32]. Special efforts were made to ensure OPRIPALC’s presence in the Latin American IRPA Congress, Santiago, Chile, 2022 [33], including the submission of contributions and the attendance of representatives from different centers.

In addition, the following scientific articles have been published in peer reviewed journals: (1) “Optimization of Protection in Pediatric Interventional Radiology and Cardiology in Latin America and the Caribbean (OPRIPALC)” [34]. (2) “Setting up regional diagnostic reference levels for pediatric interventional cardiology in Latin America and the Caribbean countries: preliminary results and identified challenges” [35].

### 3.5. Working Groups and Related Documents

A working group was established to focus on quality control (QC). It was decided to use the tests proposed in the TECDOC 1958 quality control document as basic quality control tests for interventional equipment [36]. Another working group was created to address nomenclature and classification of pediatric cardiology interventional procedures. An initial categorization has been proposed for 13 procedures taking into account their complexity and expected doses, and they have been grouped into three radiation dose levels (low, medium and high). A standard research protocol has been developed and distributed among the centers that required authorization from their corresponding ethics committees, and progress has been made on the development of a consensus document that contains practical guidance for optimizing radiological protection and safety in pediatric interventional procedures.

## 4. Discussion

This paper summarizes the development, advancements, challenges and achievements of the OPRIPALC program between the years 2018 and 2023 (June). As shown in Figure 3, we have moved from the 36 centers corresponding to 9 countries reported between the years 2018 and 2019 to the 33 centers that correspond to 13 countries, which are currently part of OPRIPALC.

One of the challenges that the OPRIPALC project had to overcome was the impact that the centers experienced during the years of the COVID-19 pandemic. Table 2 highlights that there was a significant decrease in the number of interventional procedures, favoring the most serious situations in newborn patients.

Progress is being made in achieving the proposed objectives with a progressive evaluation of the DRLs values (See Figure 2, Figure 3, Figure 4, Figure 5 and Figure 6) in pediatric interventions in this region of the world. The DRLs are classified into diagnostic and therapeutic procedures, in addition to grouping them by age and weight bands. In order to contribute to radiological culture and safety and improve the quality of care in these procedures, a generic research protocol freely available was prepared for the use of the centers requiring authorization from their ethics committees to participate in OPRIPALC [28].

The results presented in Figure 2 are based on a sample size that is too small to permit a meaningful comparison with the results depicted in Figure 4 and Figure 5. When analyzing the DRLs between Figure 4 and Figure 5, they demonstrate a clear upward trend for all age groups and types of procedures. Similarly, in the case of DRLs grouped by weight groups (see Figure 3 and Figure 6), the same increasing trend in values is observed.

Data collection is an important aspect of this activity. An Excel template was also distributed among the participating centers to manually collect patient dose values and establish the DRLs. Additionally, the Medical Physics Service of the San Carlos Hospital in Madrid facilitated the DOLQA dose management software program, http://fisica-medica.uta.cl (accessed on 1 September 2023), which was installed on a server at the University of Tarapacá (Arica, Chile) for possible connections of some of the hospitals involved in OPRIPALC to receive and process structured dose reports and generate alerts for potential optimization actions [30]. It is foreseen, as a challenge, the possibility of additionally managing occupational doses in some of the centers that could have personal electronic dosimeters as it is already being performed at the San Carlos Hospital in Madrid [29]. Currently, the Roberto del Rio Hospital in Chile is already connected with a sample of 223 procedures, and during the year 2023, it is expected that it will be also installed at the Italian Hospital in Buenos Aires, Argentina.

The OPRIPALC website has proved to be an important tool to inform and follow up the updated information of the program, including updated indicators of the number of procedures incorporated into the database, bibliography, etc. In addition, there is a password protected section where information and materials are shared only among professionals who actively participate in a center affiliated with OPRIPALC [28].

During these years of OPRIPALC activity, at least a dozen of training webinars have been conducted. Likewise, a significant number of scientific papers have been presented at congresses, seminars and technical meetings in different countries and continents [33]. It is also relevant to mention the publication of two scientific articles in indexed journals. The objective of first article was to describe the OPRIPALC program in relation to its objectives, proposed activities and expected results [34]. The second article had as objective to propose a set of preliminary regional DRLs for pediatric IC procedures for Latin America and the Caribbean countries, classified by age and weight bands [35].

To respond to one of the main objectives of the OPRIPALC program, a regional consensus guide for optimizing protection and safety in pediatric interventional procedures in Latin America and the Caribbean has been drafted. This document includes the description of technical actions to improve protection and safety in these practices within the framework of the OPRIPALC program. The objective is to provide a practical guidance to be used by health professionals working in this field. This document will address key aspects such as quality assurance and minimum quality control tests for X-ray equipment, nomenclature and classification of procedures including the evaluation of their complexity and the impact on reference levels, radiological protection programs, actions to optimize radiological protection of patients and operators during these procedures, among others.

Additionally, it is necessary to continue updating the radiological protection training of the professionals, including, among other aspects, the importance of DRLs and the communication of the benefits and risks of the use of ionizing radiation to the relatives of the patients [37]. It is also key to advance in training on occupational radiation protection. Further analysis of occupational radiation dose levels and their correlation with the complexity of the procedures and with the patient doses are needed, using thermoluminescent, optically luminous and electronic dosimetry (as it is already being performed at the San Carlos Hospital in Madrid).

It is key to continue disseminating the outcomes of the OPRIPALC program and share its experience with other countries and regions of the world. A true community has been formed around the safety and radiological protection of pediatric interventional procedures, with interventional physicians (cardiologists, radiologists, etc.), medical physicists, medical technologists and X-ray equipment maintenance engineers actively participating.

It has also been a challenge to involve the industry that manufactures medical equipment more actively in these types of international initiatives, facilitating, for example, which X-ray systems that have the option available the Digital Imaging and Communication in Medicine (DICOM) Radiation Dose Structured Reports (RDSRs), which can send dosimetric, geometric and other technical details for each radiation event to the Picture Archiving and Communication System (PACS), which represents an important advances when it comes to managing the radiation dose of patients. In our case, to use the DOLQA dose management system, it is a fundamental requirement that the X-ray systems have the RDSRs option enabled [29,30]. The manual collection of patient dose data in OPRIPALC represents a limitation of the project, but it should be overcome in the short term with the incorporation of DOLQA in more centers. The small number of centers that have implemented their quality control tests and the calculation of the correction factor to the value of kerma area-product also represent limitations.

The OPRIPALC program represents an organized international effort that can serve as a model to be applicable in other regions of the world. It is relevant to highlight that OPRIPALC was able to overcome, not without difficulties, the work and communication problems experienced globally as a result of the COVID-19 pandemic. The continuation of the OPRIPALC program, at least in a second phase, is essential to address pending issues, including the optimization of the DRLs achieved, the integration of automatic dose management systems, the determination of occupational radiation dose levels and its correlation with doses in pediatric patients.

## Figures and Tables

**Table 1:** Participating centers.

N° Center	Country	Membership	Web Site
1	ARGENTINA	Hospital Nacional de Pediatría J.P. Garrahan	www.garrahan.gov.ar (accessed on 1 September 2023)
2	ARGENTINA	Hospital Italiano	www.hospitalitaliano.org.ar (accessed on 1 September 2023)
3	ARGENTINA	Hospital de Niños Pedro de Elizalde	www.buenosaires.gob.ar (accessed on 1 September 2023)
4	ARGENTINA	Hospital de Niños Santísima Trinidad	www.moovitapp.com (accessed on 1 September 2023)
5	ARGENTINA	Instituto de cardiología de corrientes	www.icc.org.ar (accessed on 1 September 2023)
6	BRAZIL	Hospital Santa Isabel	www.hospitalsantaizabel.org.br (accessed on 1 September 2023)
7	BRAZIL	Hospital Universitário da Universidade Federal do Maranhão (HU-UFMA)	www.gov.br (accessed on 1 September 2023)
8	BRAZIL	Hospital São Paulo-hospital universitário da Universidade Federal de São Paulo.	www.hu.usp.br (accessed on 1 September 2023)
9	BRAZIL	Hospital Pequeno Príncipe	www.pequenoprincipe.org.br (accessed on 1 September 2023)
10	BRAZIL	Hospital de Clínicas da Universidade Federal do Paraná (HC-UFPR)	www.ufpr.br (accessed on 1 September 2023)
11	BRAZIL	Instituto Materno Infantil de Pernambuco (IMIP)	www1.imip.org.br (accessed on 1 September 2023)
12	BRAZIL	Pronto Socorro Cardiológico Universitário de Pernambuco (PROCAPE)	www.upe.br/procape.html (accessed on 1 September 2023)
13	BRAZIL	Real Hospital Português de Beneficência em Pernambuco (RHP)	www.rhp.com.br (accessed on 1 September 2023)
14	CHILE	Hospital Luis Calvo Mackenna	www.calvomackenna.cl/ (accessed on 1 September 2023)
15	CHILE	Hospital de Niños Roberto del Rio	www.hrrio.cl/web2 (accessed on 1 September 2023)
16	CHILE	Clínica Santa María	www.clinicasantamaria.cl (accessed on 1 September 2023)
17	CHILE	Hospital Clínico Pontificia Universidad Católica de Chile	www.ucchristus.cl (accessed on 1 September 2023)
18	COLOMBIA	Fundación Valle del Lili Hospital Universitario	www.valledellili.org (accessed on 1 September 2023)
19	COLOMBIA	Clínica IMAT Oncomédica Auna	www.imatoncomedica.com/ (accessed on 1 September 2023)
20	COSTA RICA	Hospital Nacional de Niños	www.ach.sa.cr/directorio/listado (accessed on 1 September 2023)
21	CUBA	Cardiocentro Pediátrico “William Soler”	www.dirinstituciones.sld.cu (accessed on 1 September 2023)
22	ECUADOR	Hospital Metropolitano	www.hospitalmetropolitano.org (accessed on 1 September 2023)
23	ECUADOR	Hospital de especialidades Carlos Andrade Marín	www.hcam.iess.gob.ec/ (accessed on 1 September 2023)
24	GUATEMALA	Unidad de Cirugía Cardiovascular de Guatemala (UNICAR)	www.unicargt.org (accessed on 1 September 2023)
25	MEXICO	Instituto Nacional de Cardiología Ignacio Chávez	www.cardiologia.org.mx (accessed on 1 September 2023)
26	MEXICO	Centro de Especialidades Médicas del Sureste SA de CV	www.cemsureste.com (accessed on 1 September 2023)
27	MEXICO	Hospital Zambrano Hellion	www.tecsalud.mx (accessed on 1 September 2023)
28	PANAMA	Hospital del Niño Doctor José Renán Esquivel	www.hn.sld.pa (accessed on 1 September 2023)
29	PERU	Instituto Nacional Salud del Niño San Borja	www.insnsb.gob.pe (accessed on 1 September 2023)
30	PERU	Instituto Nacional Cardiovascular (INCOR)	www.portal.essalud.gob.pe (accessed on 1 September 2023)
31	URUGUAY	Instituto de Cardiología Infantil	www.ici.org.uy (accessed on 1 September 2023)
32	VENEZUELA	Policlínica Metropolitana	www.policlinicametropolitana.org (accessed on 1 September 2023)
33	VENEZUELA	Hospital Cardiológico Infantil Latinoamericano	www.cardiologicoinfantil.gob.ve (accessed on 1 September 2023)

**Table 2:** Summary of most frequent responses about the impact of the COVID-19 pandemic.

1. Reduction in the number of interventional procedures between 50 and 96%. 2. Elective and semi-elective interventional procedures were deferred, accepting exclusively emergencies predominant in newborn patients. 3. Greater delay in interventional procedures due to the use of personal protection elements. 4. Difficulty in purchasing devices and other supplies. 5. Need to perform immunological tests before interventional procedures for the safety of staff and other patients in the centers. 6. Staff relocation. Physicians assigned to pediatric interventional cardiology were sent to intensive care units to care for COVID-19 patients.

## Data Availability

Data are contained within the article.
